# Exosomal miR‐205‐5p derived from periodontal ligament stem cells attenuates the inflammation of chronic periodontitis via targeting XBP1

**DOI:** 10.1002/iid3.743

**Published:** 2022-12-19

**Authors:** Lixun Kang, Yibin Miao, Ying Jin, Siyu Shen, Xiaoping Lin

**Affiliations:** ^1^ Department of Stomatology, Shengjing Hospital China Medical University Shenyang City Liaoning Province China

**Keywords:** chronic periodontitis, eosomes, lipopolysaccharide, microRNA‐205‐5p, XBP1

## Abstract

**Introduction:**

Chronic periodontitis (CP) is an inflammatory periodontal disease with high incidence and complex pathology. This research is aimed to investigate the function of exosomal miR‐205‐5p (Exo‐miR‐205‐5p) in CP and the underlying molecular mechanisms.

**Method:**

Exo‐miR‐205‐5p was isolated from miR‐205‐5p mimics‐transfected periodontal ligament stem cells (PDLSCs), and subsequently cocultured with lipopolysaccharide (LPS)‐induced cells or injected into LPS‐treated rats. The mRNA expression of inflammatory factors and Th17/Treg‐related factors were measured by quantitative real‐time PCR. The contents of inflammatory factors and the percentages of Th17/Treg cells were measured by enzyme‐linked immunosorbent assay and flow cytometry, respectively. Besides, the target relation between miR‐205‐5p and X‐box binding protein 1 (XBP1) was explored.

**Results:**

MiR‐205‐5p was downregulated in LPS‐induced PDLSCs and corresponding exosomes. Exo‐miR‐205‐5p inhibited inflammatory cell infiltration, decreased the production of TNF‐α, IL‐1β, and IL‐6, and decreased the percentage of Th17 cells in LPS‐treated rats. In addition, XBP1 was a target of miR‐205‐5p. Overexpression of XBP1 weakened the effects of Exo‐miR‐205‐5p on inhibiting inflammation and regulating Treg/Th17 balance in LPS‐induced cells.

**Conclusions:**

Exo‐miR‐205‐5p derived from PDLSCs relieves the inflammation and balances the Th17/Treg cells in CP through targeting XBP1.

## Graphical abstract

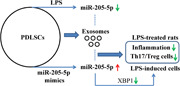

## 1 INTRODUCTION

Chronic periodontitis (CP) is a common and complex inflammatory disorder accompanied with multiple etiologies, such as bacterial infection, dental calculus, and genetic factors.^1^, ^2^, ^3^ CP occurs on specific tooth, usually at the top of the tooth without effects on adjacent tooth.^4^ The onset of CP is severe in the initial stage, followed by a long‐term stable period with multiple mild recurrences.^5^ In the world, the incidence of periodontitis increases by 57.3% from 1990 to 2010 along with an upward trend year by year.^6^ Currently, eliminating the infection and inflammation is a final goal of treatment for CP, but the clinical outcomes remain unsatisfactory.^7^ Therefore, more effective treatment methods are still required to be explored.

Exosomes are small secretory organelles containing various signal substances (miRNAs and proteins), which can release into the extracellular environment from cells.^8^ A variety of exosomal miRNAs have been reported to exert unique functions in periodontitis.^9^, ^10^, ^11^, ^12^, ^13^, ^14^ For example, miR‐140‐5p, ‐146a‐5p, and ‐628‐5p are overexpressed in small extracellular vesicles from patients with periodontitis, considering potential biomarkers.^14^ Exosomal miR‐1246 from dental pulp stem cells promotes the transition of periodontal tissues from a proinflammatory phenotype to an anti‐inflammatory phenotype.^10^ Exosomal miR‐200c decreases proinflammatory cytokines in mice with lipopolysaccharide (LPS)‐induced periodontitis.^13^ In addition, exosomal miR‐205 is an important regulator in human cancers, which can act a biomarker of lung and clear cell renal cancer^15^, ^16^ and also participates in the metastasis of ovarian cancer cells.^17^ Nevertheless, the regulatory role of exosomal miR‐205 on periodontitis is rarely known.

Th17/Treg cells derived from CD4+ T cells are important in the pathogenesis of diverse human diseases, such as type 2 diabetes,^18^ autoimmune thyroid diseases,^19^ and cancers.^20^, ^21^ Among them, Treg cells with positive FoxP3 expression are required for immune induction in inhibiting the process of inflammation.^22^ On the contrary, Th17 cells participate in inflammation via producing IL‐17, which can be activated by the interaction of TGF‐β with IL‐21, IL‐6, and transcription factor retinoic acid receptor‐related orphan receptor‐γt (RORγt).^23^ The balance of Th17/Treg cells is also of significance to the development of periodontitis.^24^, ^25^, ^26^, ^27^ For example, the increasing of Th17/Treg cells is involved in the enlargement of inflammatory periapical lesions.^25^, ^26^ IL‐35 inhibits alveolar bone resorption in periodontitis through elevating Th17/Treg cells.^27^ Upregulation of CD40 is associated with Th17/Treg imbalance in CP.^24^ Notably, periodontal ligament stem cells (PDLSCs)‐derived exosomal miR‐155‐5p alleviates inflammation in CP through regulating Th17/Treg.^28^ Moreover, a previous study revealed that miR‐205 maintains T cell development in thymus under stress.^29^ However, the role of miR‐205‐5p on Th17/Treg balance has not been studied in CP.

X‐box binding protein 1 (XBP1) is a transcription factor mediated by alternative splicing of the endoplasmic reticulum and unfolded proteins.^30^ XBP1 can be regulated by many miRNAs in inflammatory diseases. For example, overexpression of miR‐330‐3p aggravates ulcerative colitis through downregulating XBP1.^31^ MiR‐665 enhances cell apoptosis and colitis through inhibiting XBP1 in inflammatory bowel disease.^32^ Moreover, the expression of XBP1 has been confirmed to be upregulated in tissues affected by periodontitis.^33^, ^34^ Therefore, we suspect whether miR‐205‐5p functions in CP through regulating XBP1. In this study, exosomal miR‐205‐5p (Exo‐miR‐205‐5p) was isolated from miR‐205‐5p mimics‐transfected PDLSCs. The effects of Exo‐miR‐205‐5p on the inflammation and Th17/Treg balance were evaluated in a rat model of CP. The action mechanisms of Exo‐miR‐205‐5p relating XBP1 were further analyzed in LPS‐induced cells. This study is designed to discover the effects and mechanisms of Exo‐miR‐205‐5p in CP, providing potential therapeutic targets.

## 2 METHODS

### 2.1 Culturing of PDLSCs and transfection

Periodontal ligament stem cells (PDLSCs; iCell Bioscience) were cultured in DMEM (low glucose; Thermo Fisher Scientific) containing 10% FBS and 1% streptomycin/penicillin at 37°C and 5% CO_2_. PDLSCs between the third and sixth generations were used for transfection. Mimics NC/miR‐205‐5p mimics (30 nM) or pcDNA3.1‐XBP1 (Ribobio) were transfected into PDLSCs using Lipotransfectamine 3000 (Thermo Fisher Scientific) for 48 h. In addition, PDLSCs also received the treatment of 100 ng/ml LPS for 24 h to imitate the environment of PC in vitro.

### 2.2 Identification of exosomes derived from PDLSCs

Exosomes were isolated from PDLSCs and then identified as previous described.^12^ Briefly, the supernatants of PDLSCs that centrifuged at 300 × *g* for 15 min were filtered through a filter membrane (0.22 μm). After 70 min of ultracentrifugation at 100,000 × *g* and 4°C, the particles (exosomes) were collected. For identification, the diameter of isolated exosomes was measured by nanoparticle tracking analysis (NTA) on a NanoSight NS300 system (NanoSight). The morphology was observed under a transmission electron microscopy (TEM; JEM‐2100; JEOL). The positive status of exosomes for the markers of cluster of differentiation (CD)9, CD63, and tumor susceptibility gene 101 protein (TSG101) was determined by western blot.

### 2.3 Establishment of a rat model of PC

Animal experiments were approved by the ethics committee of China Medical University, strictly in accordance with the Guidelines for the Care and Use of Laboratory Animals. Male Sprague‐Dawley rats (SPF grade, 8 weeks old, 300 ± 15 g; Laboratory Animal Center of China Medical University) were routinely fed in a room at 25°C, 55%−65% humidity, and 12 h light cycle. For treatments, rats were randomly divided into three groups, including control, LPS, and LPS + Exo‐miR‐205‐5p groups (*N* = 6 each group). In the LPS group, 10 μl *Escherichia coli* LPS (2 mg/ml; Sigma‐Aldrich) was injected into the gingival sulcus between the first and second molars every 2 days.^35^ Rats in the LPS + Exo‐miR‐205‐5p group were injected with 10 μg Exo‐miR‐205‐5p (exosomes isolated from miR‐205‐5p mimics‐transfected PDLSCs) following consistent LPS treatment every 2 days. In addition, rats injected with the same amount of normal saline were enrolled in the control group. After 4 weeks of treatments, rats were anesthetized with 2% pentobarbital sodium (50 mg/kg; Sigma‐Aldrich), and the blood was collected from the heart. Subsequently, rats were immediately killed with excessive pentobarbital sodium, and the gingival tissues surrounding the injection area were collected for the following assays.

### 2.4 Hematoxylin and eosin (HE) staining

The resected gingival tissues in the same anatomic site were sequentially fixed in 4% formalin, demineralized in 10% disodium ethylenediaminetetraacetic acid, dehydrated, paraffin‐embedded, and sliced at 5 μm. Subsequently, the sections were dewaxed, rehydrated, and stained with hematoxylin for 4 min and eosin (Beyotime) for 2 min. The inflammatory cell numbers were counted under a microscope (CKX53; Olympus) at three randomly selected fields.

### 2.5 Flow cytometry

Treg/Th17 cells was detected by flow cytometry as previously described.^36^ Simply, blood lymphocytes were isolated from blood samples via gradient centrifugation, and CD4+ T cells were then separated from lymphocytes using CD4 microbeads (NovoBio). Cells positive for IL‐10 and Foxp3 (Treg cells), or IL‐17A and RoR‐γτ (Th17 cells) were detected on a flow cytometer (Becton Dickinson). In addition, CD4+ T cells isolated from the control rats also received the treatment of 0.1 mg/ml LPS, Exo‐miR‐205‐5p, and/or pcDNA3.1‐XBP1 (Ribobio). Treg/Th17 balance in these cells was also detected.

### 2.6 Quantitative real‐time PCR (qRT‐PCR)

The RNA samples in cells or tissues were extracted using Trizol (Thermo Fisher Scientific) and quantified on an ultramicro spectrophotometer (Nanodrop2000; Thermo Fisher Scientific). cDNA was reverse transcripted using PrimeScript RT kit (Takara) for detecting mRNAs or using miRNA First‐Strand Synthesis kit (Takara) via poly(A)‐tailed method for detecting miR‐205‐5p. qRT‐PCR was run on a QuantStudio7 system (Thermo Fisher Scientific) using SYBR Green Premix (Thermo Fisher Scientific) and specific primers (Table 1). The conditions included an initial 95°C for 10 min, and 40 cycles of 95°C for 15 s and 60°C for 30 s. The expression level was calculated by the $2^{-\Delta \Delta C_{t}}$method. U6 and β‐actin were used as internal controls (U6 for miR‐205‐5p and β‐actin for other genes).

**Table 1:** Primer sequence for qRT‐PCR

Gene	Sequence (5′‐3′)
miR‐205‐5p	F: TCCTTCATTCCACCGGAGTCTG
	R: GCGAGCACAGAATTAATACGAC
U6	F: AGAGCCTGTGGTGTCCG
	R: CATCTTCAAAGCACTTCCCT
β‐actin	F: CGGGACCTGACTGACTACCTC
	R: CCATCTCTTGCTCGAAGTCCAG
XBP1	F: ACATCTTCCCATGGACTCTG
	R: TAGGTCCTTCTGGGTAGACC
TNF‐α	F: AGGACACCATGAGCACTGAAAGC
	R: AAGGAGAAGAGGCTGAGGAACAAG
IL‐1β	F: TGTGAAATGCCACCTTTTGA
	R: TGAGTGATACTGCCTGCCTG
IL‐6	F: TACCACTTCACAAGTCGGAGGC
	R: CTGCAAGTGCATCATCGTTGTTC
RORγτ	F: AGTGTAATGTGGCCTACTCCT
	R: GCTGCTGTTGCAGTTGTTTCT
IL‐17A	F: GAGAAGATGCTGGTGGGT
	R: TTTGCTGAGAAACGTGGG
Foxp3	F: CAAGGAAAGGAGGATGGACGAACA
	R: TGGCAGGCAAGACAGTGGAA
IL‐10	F: GTGATGCCCCAAGCTGAGA
	R: CACGGCCTTGCTCTTGTTTT

Abbreviations: qRT‐PCR, quantitative real‐time PCR; XBP, X‐box binding protein.

### 2.7 Enzyme‐linked immunosorbent assay (ELISA)

The contents of IL‐6, IL‐1β, and TNF‐α were measured in the serum of LPS‐treated rats and also in LPS‐induced PDSLCs. This experiment was performed using specific ELISA kits (Esebio) in accordance with the instructions.

### 2.8 Dual luciferase reporter (DLR) assay

Candidate target genes of miR‐205‐5p were predicted by Starbase (v2.0, https://starbase.sysu.edu.cn/index.php). DLR was performed to confirm the target relationship between miR‐205‐5p and XBP1. Simply, the fragments of XBP1 wild‐type (XBP1‐wt) and XBP1 mutant (XBP1‐mut) were integrated into luciferase vector (Promega). PDLSCs were then cotransfected with recombinant vectors and miR‐205‐5p mimics/mimics NC for 48 h. DLR assay kit (Promega) was used to measure the luciferase activity.

### 2.9 RNA immunoprecipitation (RIP) assay

RIP assay was performed to confirm the target relationship using RIP kit (Millipore). Simply, the lysed PDLSCs were incubated with IgG or anti‐Ago2 beads for 12 h. The miR‐205‐5p and XBP1 expression were detected in immunoprecipitated RNAs via qRT‐PCR.

### 2.10 RNA pull‐down assay

The target relationship was also identified by RNA pull‐down assay. Simply, the PDLSCs were transfected with biotin‐labled NC‐Bio, miR‐205‐5p‐Bio, and miR‐205‐5p‐mut‐Bio (GenePharma), respectively. After the transfection for 24 h, cell lysates were incubated with Streptavidin‐coupled dynabeads (Thermo Fisher Scientific) for 1 h. The expression of XBP1 was detected in the eluents by qRT‐PCR.

### 2.11 Western blot

Cells were lysed in RIPA lysis buffer (Beyotime) to isolate total proteins. The proteins were separated by SDS‐PAGE, transferred onto PVDF membranes (Millipore), blocked with 5% nonfat milk for 1 h, and incubated with primary antibodies for 12 h. After continuous incubated with secondary antibody (1:2000; Abcam) for 2 h, the blots were visualized using ECL kit (Beyotime). The primary antibodies included anti‐CD9 (1:2000; Abcam), ‐CD90 (1:1000; Abcam), ‐CD63 (1:1000; Abcam), ‐CD105 (1:1000; Abcam), ‐TSG101 (1:500; Santa Cruz), ‐XBP1 (1:2000; Santa Cruz), and ‐GAPDH (1:5000; Abcam).

### 2.12 Statistical analyses

Data were presented as mean ± standard deviation, and statistical analyzed by SPSS 22.0. Student's *t‐*test was performed to compare the differences between two groups. One‐way ANOVA followed by Tukey's test was performed to compare the differences among multiple groups. A *p* < .05 represented significantly different.

## 3 RESULTS

### 3.1 Identification of PDLSC‐derived exosomes

**Figure 1:**
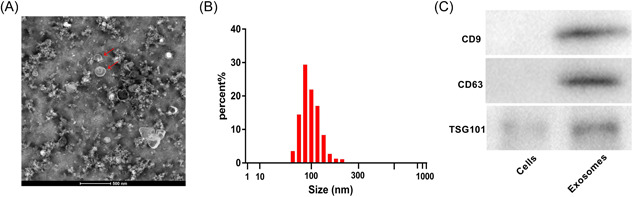
Identification of exosomes derived from PDLSCs. (A) The morphology of exosomes was observed under TEM (bar = 500 nm). (B) The diameter distribution of exosomes was determined by NTA. (C) The protein expression of CD9, CD63, and TSG101 in exosomes and PDLSCs were detected by western blot. NTA, nanoparticle tracking analysis; PDLSCs, periodontal ligament stem cells; TSG101, tumor susceptibility gene 101 protein.

The exosomes that isolated from PDLSCs were saucer‐shaped under TEM (Figure 1A). NTA revealed that the diameter of PDLSC‐derived exosomes was approximately 50−120 nm (Figure 1B). In addition, PDLSC‐derived exosomes were determined to be positive for TSG101, CD9, and CD63 (Figure 1C).

### 3.2 MiR‐205‐5p is downregulated in LPS‐induced PDLSCs and corresponding exosomes

**Figure 2:**
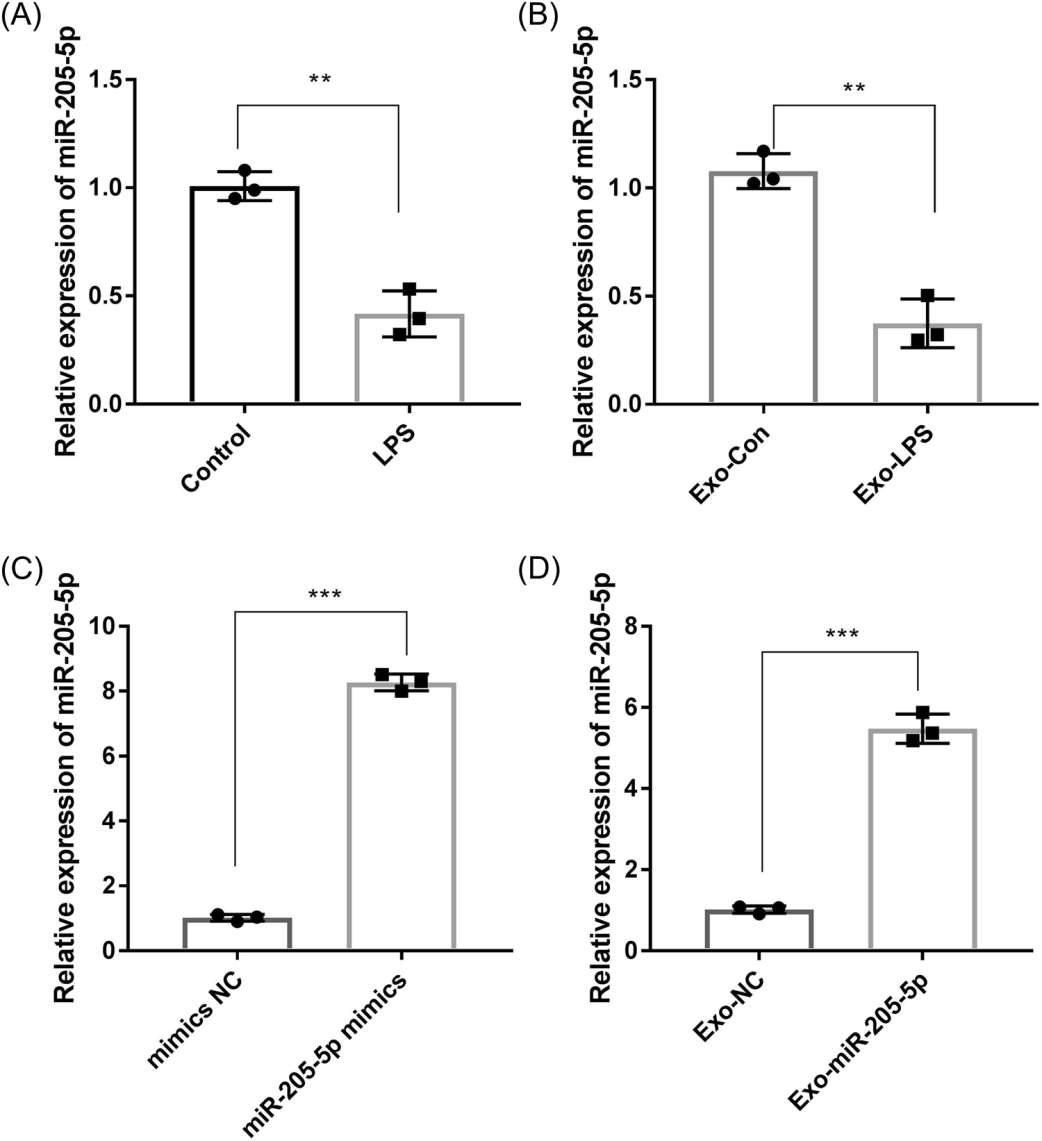
MiR‐205‐5p is downregulated in LPS‐induced PDLSCs and corresponding exosomes. (A) The expression of miR‐205‐5p in LPS‐treated PDLSCs and controls was detected by qRT‐PCR. (B) The expression of miR‐205‐5p in exosomes derived from LPS‐treated PDLSCs (Exo‐LPS) and controls (Exo‐Con) was detected by qRT‐PCR. (C) The expression of miR‐205‐5p in PDLSCs transfected with miR‐205‐5p mimics and mimics NC was detected by qRT‐PCR. (D) The expression of miR‐205‐5p in exosomes derived from miR‐205‐5p mimics/mimics NC‐transfected PDLSCs (Exo‐miR‐205‐5p and Exo‐NC) was detected by qRT‐PCR. Three independent experiments were performed in triplicates. **p < .01, ***p < .001. LPS, lipopolysaccharide; PDLSCs, periodontal ligament stem cells; qRT‐PCR, quantitative real‐time PCR.

The expression of miR‐205‐5p was detected in LSP‐induced PDLSCs and corresponding exosomes by qRT‐PCR. The treatment of LPS significantly downregulated miR‐205‐5p in PDLSCs and also in corresponding exosomes (*p* < .01, Figure 2A,B). MiR‐205‐5p was then overexpressed in PDLSCs to evaluate the function of Exo‐miR‐205‐5p in CP. Compared with PDLSCs transfected with mimics NC, miR‐205‐5p was significantly upregulated in PDLSCs transfected with miR‐205‐5p mimics (*p* < .001, Figure 2C). A higher miR‐205‐5p expression was also revealed in the Exo‐miR‐205‐5p group than the Exo‐NC group (*p* < .001, Figure 2D).

### 3.3 Exo‐miR‐205‐5p relieves the inflammation in LPS‐treated rats

**Figure 3:**
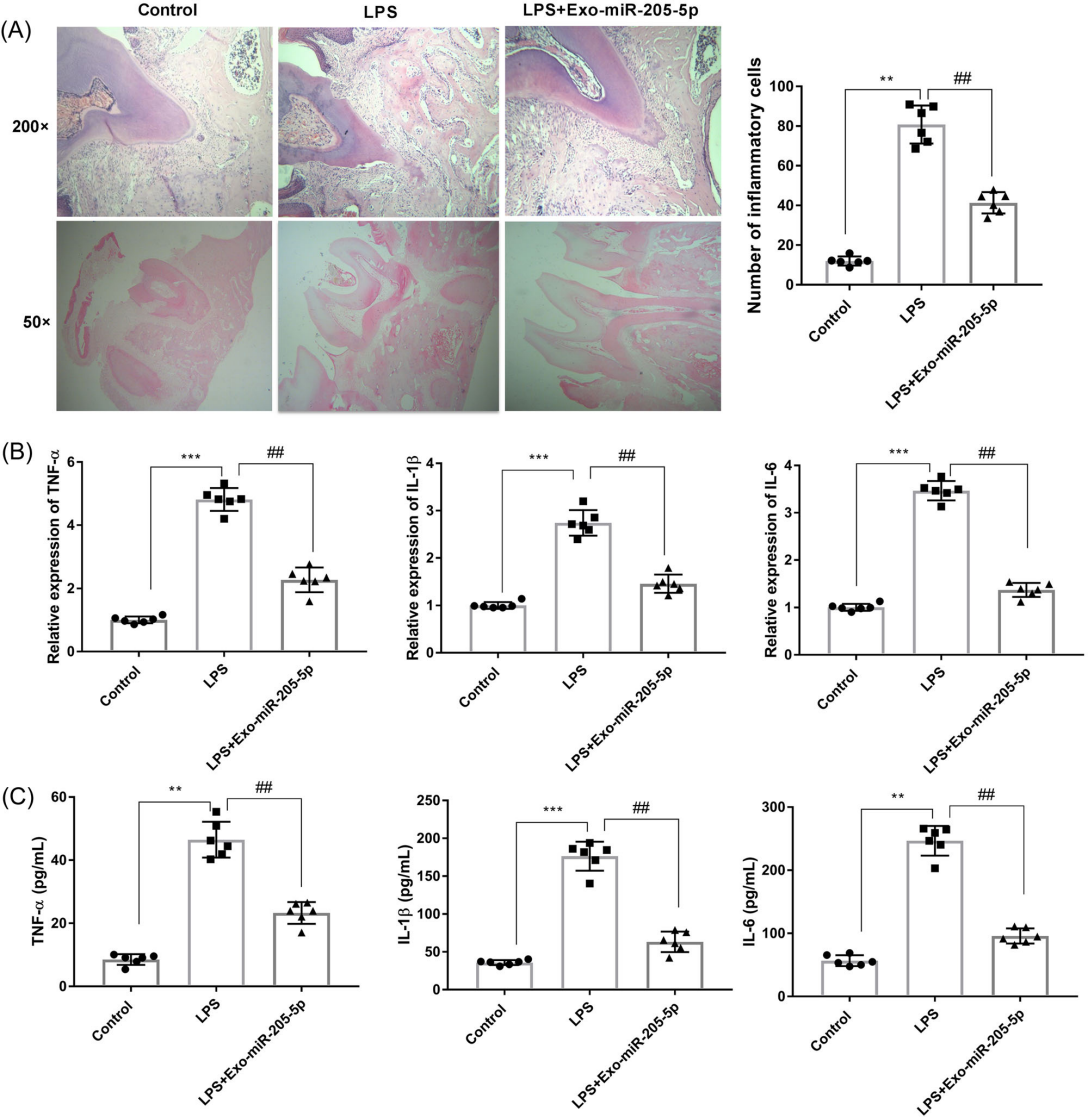
Exo‐miR‐205‐5p inhibits the inflammation in a rat model of CP. (A) The inflammatory cell infiltration in gingival tissues of the same anatomic site was detected by HE staining (×200 and ×50). (B) The mRNA expression of TNF‐α, IL‐1β, and IL‐6 in gingival tissues was detected by qRT‐PCR. (C) The serum levels of TNF‐α, IL‐1β, and IL‐6 were detected by ELISA. Rats were injected with normal saline (control), LPS, and LPS + Exo‐miR‐205‐5p (*N* = 6 each group). Each experiment was performed in triplicates **p < .01, ***p < .001, ^##^ *p* < .01. CP, chronic periodontitis; ELISA, enzyme‐linked immunosorbent assay; LPS, lipopolysaccharide; qRT‐PCR, quantitative real‐time PCR.

Since CP is accompanied with obvious inflammation, the anti‐inflammatory potential of Exo‐miR‐205‐5p was evaluated in LPS‐treated rats. HE staining showed more inflammatory cells in the LPS group in comparison with the control group (*p* < .01). Exo‐miR‐205‐5p weakened the increasing of inflammatory cells that induced by LPS (*p* < .01, Figure 3A). Besides, the expression of TNF‐α, IL‐1β, and IL‐6 at the mRNA level were significantly increased in the LPS group in comparison with the control group (*p* < .001). Exo‐miR‐205‐5p weakened the effects of LPS on upregulating these inflammatory factors (*p* < .01, Figure 3B). Consistent results were revealed in the serum levels of related inflammatory factors in LPS‐treated rats (*p* < .01, Figure 3C).

### 3.4 Exo‐miR‐205‐5p influences Th17/Treg balance in LPS‐treated rats

**Figure 4:**
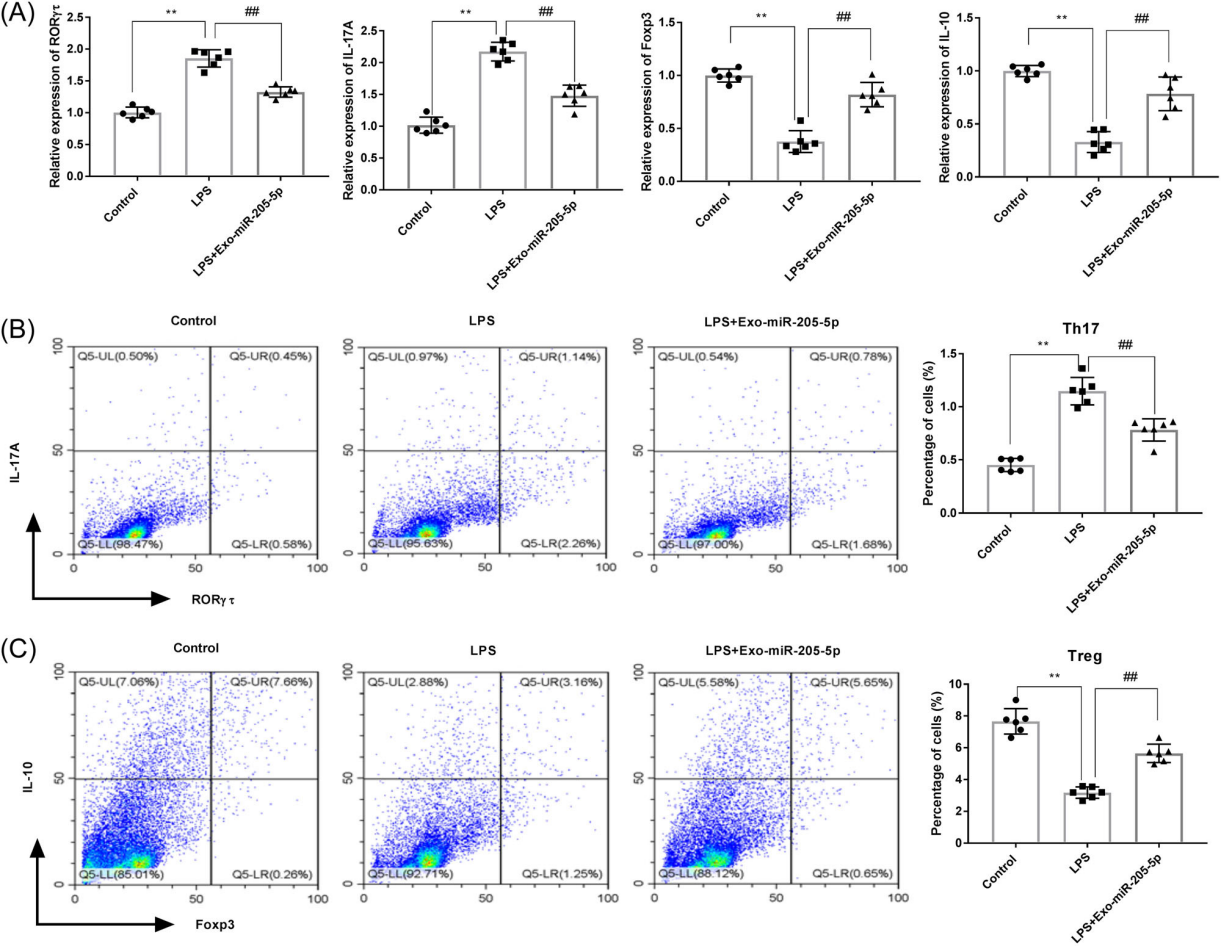
Exo‐miR‐205‐5p influences Th17/Treg balance in a rat model of CP. (A) The mRNA expression of RORγτ, IL‐17A, Foxp3, and IL‐10 in gingival tissues was detected by qRT‐PCR. (B) The percentage of Th17 cells (positive for IL‐17A and RORγτ) in blood was detected by flow cytometry. (C) The percentage of Treg cells (positive for IL‐10 and Foxp3) in blood was detected by flow cytometry. Rats were injected with normal saline (control), LPS, and LPS + Exo‐miR‐205‐5p (*N* = 6 each group). Each experiment was performed in triplicates. **p < .01, ^##^ *p* < .01. LPS, lipopolysaccharide; qRT‐PCR, quantitative real‐time PCR.

The mRNA expression of RORγτ, IL‐17A, Foxp3, and IL‐10 were measured in gingival tissues of rats in different groups. qRT‐PCR showed significantly higher RORγτ and IL‐17A expression, and lower Foxp3 and IL‐10 expression in the LPS group in comparison with the control group (*p* < .01). Exo‐miR‐205‐5p reversed the effects of LPS on the expression of these factors (*p* < .01, Figure 4A). Subsequently, the percentages of Th17 (IL‐17A, RORγτ) and Treg (IL‐10, Foxp3) cells were analyzed. Flow cytometry showed that LPS treatment increased Th17 cells and decreased Treg cells (*p* < .01). The effects of LPS on the imbalance of Th17/Treg cells were also partially eliminated by Exo‐miR‐205‐5p (*p* < .01, Figure 4B,C).

### 3.5 XBP1 is a target gene of miR‐205‐5p

**Figure 5:**
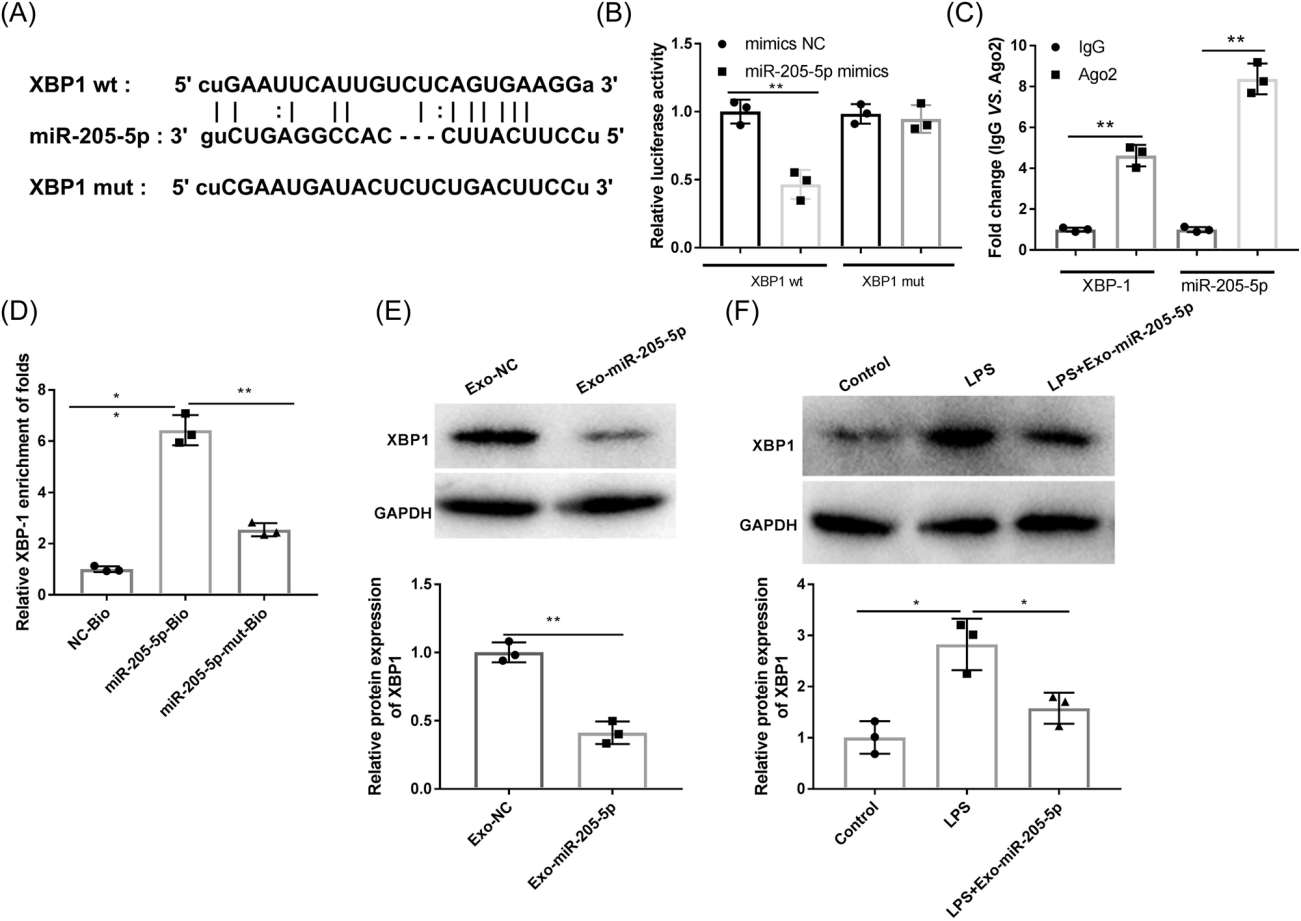
XBP1 is a target of miR‐205‐5p. (A) A binding site of miR‐205‐5p on XBP1 was predicted by Starbase. (B) DLR assay was performed to identify the target relationship. (C) RIP assay was performed to identify the target relationship. (D) RNA pull down assay was performed to identify the target relationship. (E) The protein expression of XBP1 in exosomes derived from miR‐205‐5p mimics/mimics NC‐transfected PDLSCs (Exo‐miR‐205‐5p and Exo‐NC) was detected by western blot. (F) The protein expression of XBP1 in PDLSCs treated with LPS or LPS + Exo‐miR‐205‐5p was detected by western blot. Each experiment was performed in triplicates. *p < .05, **p < .01. DLR, dual luciferase reporter; LPS, lipopolysaccharide; PDLSCs, periodontal ligament stem cells; RIP, RNA immunoprecipitation; XBP, X‐box binding protein.

There a binding site of miR‐205‐5p on XBP1 was predicated by StarBase (Figure 5A). DLR assay revealed a lower luciferase activity in the XBP1‐wt + miR‐205‐5p mimics group than the XBP1‐wt + mimics NC group (*p* < .01, Figure 5B). RIP assay found that both miR‐205‐5p and XBP1 were enriched in the Ago2 group (*p* < .01, Figure 5C). RNA pull‐down assay further indicated that XBP1 was significantly enriched in the miR‐205‐5p‐Bio group in comparison with both the NC‐Bio and miR‐205‐5p‐mut‐Bio groups (*p* < .01, Figure 5D). Besides, the protein expression of XBP1 was significantly lower in the Exo‐miR‐205‐5p group than the Exo‐NC group (*p* < .01, Figure 5E). In LPS‐induced cells, Exo‐miR‐205‐5p also significantly decreased XBP1 expression (*p* < .05, Figure 5F).

### 3.6 Exo‐miR‐205‐5p inhibits the inflammation of LPS‐induced PDSLCs through targeting XBP1

**Figure 6:**
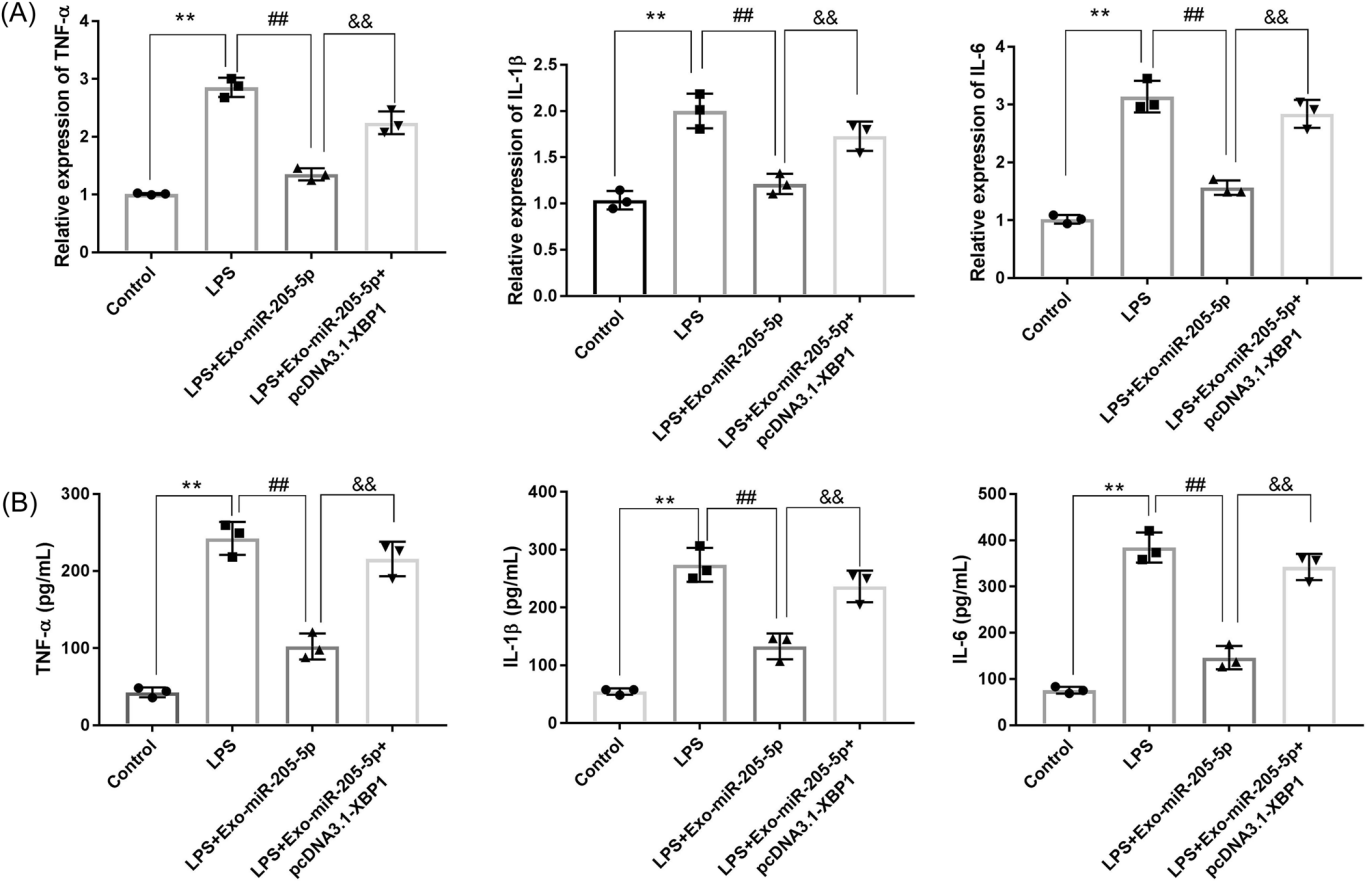
Exo‐miR‐205‐5p inhibits the inflammation of LPS‐induced PDSLCs through targeting XBP1. (A) The mRNA expression of TNF‐α, IL‐1β, and IL‐6 were detected by qRT‐PCR. (B) The levels of TNF‐α, IL‐1β, and IL‐6 were detected by ELISA. PDLSCs were treated with LPS, LPS + Exo‐miR‐205‐5p, or LPS + Exo‐miR‐205‐5p + pcDNA3.1‐XBP1. Three independent experiments were performed in triplicates. **p < .01, ^##^ *p* < .01, ^&&^ *p* < .01. ELISA, enzyme‐linked immunosorbent assay; LPS, lipopolysaccharide; PDLSCs, periodontal ligament stem cells; qRT‐PCR, quantitative real‐time PCR; XBP, X‐box binding protein.

The action mechanisms of Exo‐miR‐205‐5p on inflammation were analyzed in LPS‐induced PDSLCs. As presented in Figure 6A, EXO‐MIR‐205‐5P decreased TNF‐α, IL‐1β, and IL‐6 expression in LPS‐induced PDSLCs (*p* < .01). Overexpression of XBP1 partially reversed the inhibiting effects of Exo‐miR‐205‐5p on the expression of these inflammatory factors (*p* < .01). The contents of TNF‐α, IL‐1β, and IL‐6 in cell supernatants showed consistent results with mRNA expression (*p* < .01, Figure 6B).

### 3.7 Exo‐miR‐205‐5p influences Th17/Treg balance through targeting XBP1

**Figure 7:**
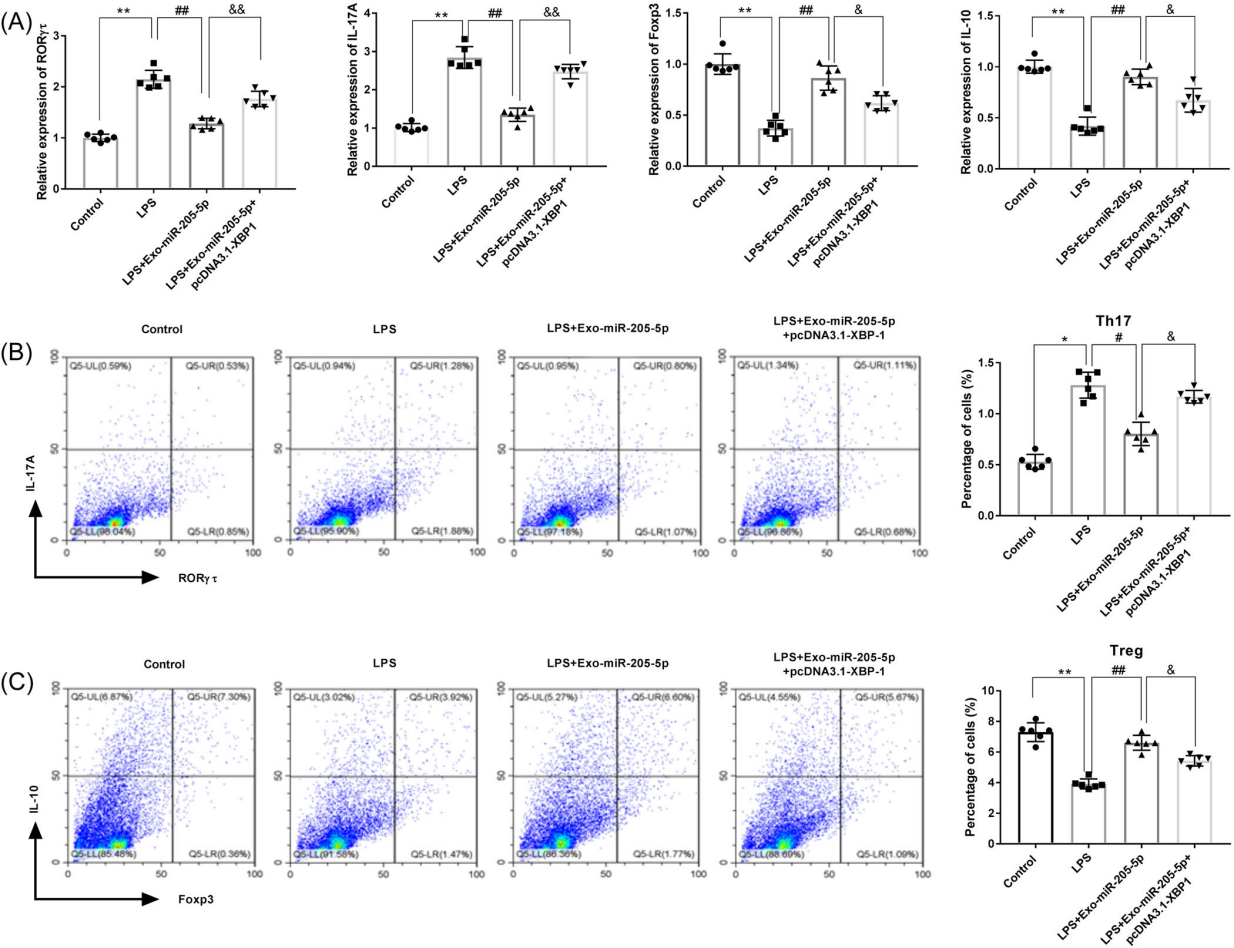
Exo‐miR‐205‐5p mediates Th17/Treg balance in LPS‐induced CD4+ T cells through targeting XBP1. (A) The mRNA expression of RORγτ, IL‐17A, Foxp3, and IL‐10 was detected by qRT‐PCR. (B) The percentage of Th17 cells (positive for IL‐17A and RORγτ) was detected by flow cytometry. (C) The percentage of Treg cells (positive for IL‐10 and Foxp3) was detected by flow cytometry. CD4+ T cells isolated from rats were treated with LPS, LPS + Exo‐miR‐205‐5p, or LPS + Exo‐miR‐205‐5p + pcDNA3.1‐XBP1 (*N* = 6 each group). Each experiment was performed in triplicates. *p < .05, **p < .01, ^#^ *p* < .05, ^##^ *p* < .01, ^&^ *p* < .05, ^&&^ *p* < .01. LPS, lipopolysaccharide; qRT‐PCR, quantitative real‐time PCR; XBP, X‐box binding protein.

The action mechanisms of Exo‐miR‐205‐5p on Th17/Treg balance were further analyzed in LPS‐induced CD4+ T cells. As presented in Figure 7A, EXO‐MIR‐205‐5P downregulated RORγτ and IL‐17A, and upregulated Foxp3 and IL‐10 in LPS‐induced CD4+ T cells (*p* < .01). Exo‐miR‐205‐5p also significantly decreased Th17 cells and increased Treg cells (*p* < .05, Figure 7B). What is important, overexpression of XBP1 partially eliminated the effects of Exo‐miR‐205‐5p on balancing Th17/Treg cells under LPS treatment (*p* < .05, Figure 7A,B).

## 4 DISCUSSION

CP is a severe public health problem, presenting similar inflammatory characteristics with some other types of chronic inflammation.^2^ The conventional strategies for the treatment of CP mainly include tooth cleaning, root planning, and conservative periodontal surgery. However, the treatment outcomes are still unsatisfactory due to the failure of throughout root cleaning, implying the need of new therapeutic strategies.^37^ Research have shown that some differentially expressed miRNAs participate in the pathogenesis of periodontitis.^38^, ^39^ For instance, miR‐92 is downregulated in periodontal diseases, which is a biomarker of periodontitis in human.^40^ MiR‐129‐5p is also downregulated in periodontitis, which plays a regulatory role in inhibiting macrophage infiltration, apoptosis, inflammation, and oxidative stress.^41^ In this research, miR‐205‐5p was downregulated in LPS‐treated PDLSCs, indicating a therapeutic potential for CP.

Exosomes play a key role in substance exchange and signal communication among cells, presenting diagnostic and therapeutic potential for human diseases.^42^ In periodontitis, miR‐103a‐3p, ‐126‐3p, and ‐150‐5p are downregulated in plasma exosomes, considering candidate biomarkers.^9^ MiR‐155‐5p expression is decreased in exosomes derived from LPS‐induced PDLSCs, which participates in CP progression.^28^ In consistent with that in LPS‐treated PDLSCs, miR‐205‐5p was also downregulated in exosomes derived from LPS‐treated PDLSCs in this study. There emerging studies have proved the crucial roles of exosomal miRNAs in inflammatory diseases. Exosomal miR‐25‐3p from peripheral blood platelet suppresses the inflammation of coronary vascular endothelial cells via reducing the levels of TNF‐α, IL‐6, and IL‐1β.^43^ Exosomal miR‐183 derived from breast cancer cells elevates the levels of TNF‐α, IL‐6, and IL‐1β in macrophages.^44^ Exosomal miR‐200c alleviates the inflammation of periodontitis via reducing IL‐6 and IL‐8.^13^ Exosomes carrying down‐expressed miR‐223 from salivary enhances IL‐1β and IL‐6 expression in periodontitis.^45^ In this research, Exo‐miR‐205‐5p was exerted from miR‐205‐5p mimic‐transfected PDLSCs and its function in CP was evaluated in LPS‐treated rats. We found that Exo‐miR‐205‐5p weakens inflammatory cell infiltration, and decreases the production of TNF‐α, IL‐6, and IL‐1β in LPS‐treated rats. These results confirm that Exo‐miR‐205‐5p can alleviate the inflammation of LPS‐induced CP. In addition, Th17/Treg balance is closely associated with inflammatory process. Some exosomal miRNAs also participate in the inflammatory response of diseases through regulating Th17/Treg balance. Exosomal miR‐29a‐3p and miR‐21‐5p increase Treg cells and decrease Th17 cells in ovarian cancer.^46^ Exosomal miR‐1246 relieves liver ischemia‐reperfusion injury through reducing Th17 and elevating Treg cells.^47^ Exosomal miR‐155‐5p acts as a regulator in Th17 reduction and Treg elevation in CP.^28^ In this study, Exo‐miR‐205‐5p increased Treg cells and decreased Th17 cells and in LPS‐treated rats. This result indicates that Exo‐miR‐205‐5p can inhibit the inflammation of CP through regulating Th17/Treg balance.

XBP1 has been reported to be upregulated in the gingival tissues of CP patients.^33^, ^34^ XBP1 is also involved diverse inflammation‐related diseases via acting a target gene of certain miRNAs.^31^, ^48^, ^49^ For example, miR‐330‐3p worsens ulcerative colitis through downregulating XBP1.^31^ MiR‐93 alleviates the inflammation of retinal epithelial cells via targeting XBP1.^48^ Downregulation of miR‐665 relieves colitis in inflammatory bowel disease through inhibiting XBP1.^49^ In our study, XBP1 was identified to be a target of miR‐205‐5p. XBP1 overexpression partially reversed the function of Exo‐miR‐205‐5p on inhibiting the generation of inflammatory factors, and on balancing Th17/Treg cells in vitro. These findings illustrate that Exo‐miR‐205‐5p inhibits the inflammation of CP through targeting XBP1.

Our study indeed has limitations. For instance, no clinical data are included to reflect the roles of miR‐205‐5p and XBP1 in CP. The evidence on the action mechanisms of Exo‐miR‐205‐5p/XBP1 in CP rats is insufficient. In addition to XBP1, there still many other targets of Exo‐miR‐205‐5p are involved in the progression of CP. Therefore, further research on these limitations are still needed.

## 5 CONCLUSION

In conclusion, Exo‐miR‐205‐5p weakens the inflammation of CP in a rat model via inhibiting the production of inflammatory factors and the imbalance of Th17/Treg cells. XBP1 is a target of miR‐205‐5p, which is involved in the action mechanisms of Exo‐miR‐205‐5p in CP. Exo‐miR‐205‐5p may be introduced as a potential therapeutic target for CP.

## AUTHOR CONTRIBUTIONS

Lixun Kang was responsible for the design and writing of the first draft, project management. Yibin Miao and Ying Jin carried out experimental operation and data analysis. Siyu Shen was mainly responsible for the investigation, collecting experimental materials, and revising important contents of the article. Xiaoping Lin carried out the investigation and research, editing and revising the manuscript, funding application, and administrative support. All authors reviewed the manuscript.

## CONFLICT OF INTEREST

The authors declare no conflict of interest.

## ETHICS STATEMENT

The experimental procedures were strictly in accordance with the principles of the Basel Declaration and the Guidelines for the Care and Use of Laboratory Animals Established by United States National Institutes of Health. Meanwhile, this study was approved by the ethics committee of China Medical University (No.0PS079K).

## Data Availability

The data that support the findings of this study are available from China Medical University.
